# The Potential of Dosimetry and the Visualization of Microbeam Arrays in NIPAM Gel at the PETRA III Synchrotron

**DOI:** 10.3390/gels11100814

**Published:** 2025-10-10

**Authors:** Thomas Breslin, Malin Kügele, Vincent de Rover, Stefan Fiedler, Tobias Lindner, Johannes Klingenberg, Guilherme Abreu Faria, Bernd Frerker, Frank Nuesken, Sofie Ceberg, Crister Ceberg, Michael Lerch, Guido Hildebrandt, Elisabeth Schültke

**Affiliations:** 1 Department of Oncology, Clinical Sciences, Lund University, 22185 Lund, Sweden; thomas.breslin@med.lu.se; 2 Department of Radiooncology, Rostock University Medical Center, 18059 Rostock, Germany; malin.kuegele@med.uni-rostock.de (M.K.); bernd.frerker@med.uni-rostock.de (B.F.); frank.nuesken@med.uni-rostock.de (F.N.); guido.hildebrandt@med.uni-rostock.de (G.H.); 3 Centre of Medical Radiation Physics, University of Wollongong, Wollongong 2522, Australia; vdr@uowmail.edu.au (V.d.R.); mlerch@uow.edu.au (M.L.); 4 European Molecular Biology Laboratory (EMBL), Hamburg Outstation, 22607 Hamburg, Germany; sfiedler@embl-hamburg.de; 5 Core Facility Multimodale Kleintierbildgebung, Rostock University Medical Center, 18055 Rostock, Germany; tobias.lindner@med.uni-rostock.de; 6 Helmholtz-Zentrum Hereon, 21502 Hamburg, Germany; johannes.klingenberg@hereon.de (J.K.); guilherme.abreu@hereon.de (G.A.F.); 7 Medical Radiation Physics, Clinical Sciences, Lund University, 22185 Lund, Sweden; sofie.ceberg@med.lu.se (S.C.); crister.ceberg@med.lu.se (C.C.)

**Keywords:** 3D visualization, beam geometry, dosimetry, microbeam radiotherapy (MRT), MRI, NIPAM gel, spatially fractionated radiotherapy

## Abstract

Spatially fractionated radiotherapy (SFRT) is emerging as a powerful tool in cancer therapy for patients who are ineligible for treatment with clinically established irradiation techniques. Microbeam radiotherapy (MRT) is characterized by spatial dose fractionation in the micrometre range. This presents challenges in both treatment planning and dosimetry. While a dosimetry system with a spatial resolution of 10 µm and an option for real-time readout already exists, this system can only record dose in a very small volume. Thus, we are exploring dosimetry in an N-isopropylacrylamide (NIPAM) gel as an option for 3D dose visualization and, potentially, also three-dimensional dosimetry in larger volumes. In the current study, we have recorded the geometric patterns of single- and multiport irradiation with microbeam arrays in NIPAM gel. Data for 3D dose distribution was acquired in a 7T small animal MRI scanner. We found that the resolution of the gel is well suited for a detailed 3D visualization of microbeam patterns even in complex multiport geometries, similar to that of radiochromic film, which is well established for recording 2D dose distribution in MRT. The results suggest that a dose–response calibration is required for reliable quantitative dosimetry.

## 1. Introduction

Modern radiotherapy techniques and the introduction of the Nobel Prize-winning checkpoint inhibitors into clinical practice have significantly increased survival and quality of life for many patients with cancers previously considered difficult to treat [1,2,3]. Unfortunately, patients with malignant brain tumours appear not to benefit from the checkpoint inhibitors. However, the results of a recent veterinary trial conducted at the European Synchrotron Radiation Facility (ESRF) in France suggest that one of the new spatially fractionated radiotherapy (SFRT) techniques, called microbeam radiotherapy (MRT), contributes to an improved quality of life and, potentially, also to an improved survival of patients with brain cancer [4,5]. After only a single microbeam irradiation session, dogs treated for brain cancer dramatically improved clinically and displayed an average tumour volume reduction between 53.8% and 69.0%, as seen in follow-up MRIs at 1 and 3 months after radiotherapy, respectively. Although MRT still requires a synchrotron facility, the development of a synchrotron-independent compact X-ray source, producing a sufficiently high photon flux and collimation for MRT, is already at an advanced stage. This compact source is based on a line focus X-ray tube, a novel concept for producing high-brilliance X-rays [6,7,8].

MRT is characterized by spatial dose fractionation in the micrometre range. A multislit collimator, inserted into a highly collimated incident beam with a high photon flux, generates a non-homogeneous irradiation field with alternating peak (high dose) and valley (low-dose) zones. An ultra-high dose rate, similar to FLASH radiotherapy (>40 Gy/s, compared to the 6–10 Gy/min typical of clinically established radiotherapy) is characteristic of the peak dose zones. MRT, both at high peak dose rates and in the absence of them, has been shown to improve tumour control compared to broad beam irradiation [9,10].

Dosimetry and planning of MRT present several challenges due to the extremely high spatial resolution required. The first challenge is associated with the technical equipment used to measure dose. Dosimeters used in clinical radiotherapy are designed for signal collection with a spatial resolution of millimetres, while microbeam dose needs to be characterized with a spatial resolution at the micrometre range, i.e., in fields smaller by three orders of magnitude. This challenge can be met by the X-Tream dosimetry system developed at the University of Wollongong in Australia [11,12,13,14]. The X-Tream system offers a high temporal resolution with 1 MHz sampling rate and a real-time readout option, along with silicon strip detectors with a spatial resolution of 10 μm. The second challenge refers to the spatial visualization of dose distribution, which is an integral component of treatment plans generated in clinical radiotherapy.

For 2D measurements, radiochromic films with high spatial resolution are frequently used to record geometrical dose distribution patterns and, in some studies, also for dosimetry purposes in MRT [15,16,17]. While these films are a well-established standard for their high accuracy and dose-rate independence, they are limited to planar dose measurements.

An important challenge in dosimetry for complex spatially fractioned radiotherapy is the need for accurate 3D volumetric analysis. Several methods have been explored to address this need, including PRESAGE gel dosimeters combined with optical CT [18] and laser fluorescence microscopy [19]. While the results of both studies are visually appealing, both methods have limitations, particularly for sample sizes typical of clinical irradiation targets, such as malignant tumours. The optical CT study was found unsuitable to determine the peak-to-valley dose ratio (PVDR), one of the most critical parameters in MRT, with unreliability increasing as PVDR values increase. The authors of the study cited a lack of true spatial resolution as the reason for the significant underestimation of the PDVR using optical CT (as low as 30% of the expected values), a known limitation of optical scanning systems dealing with steep gradients. Moreover, the availability of optical CT systems is highly limited as this is not a state-of-the-art clinical technique. Confocal microscopy, on the other hand, was able to deliver extremely high spatial resolution, providing a solid basis for realistic PVDR assessment. However, due to the limited penetration depth of confocal microscopy, this method is not practical for volumetric analysis of real-life tumour samples.

Chemically, the PRESAGE gel falls into the category of solid plastic dosimeters. They require a complex manufacturing process, compared to simpler gel dosimeters such as Fricke gels or polymer-based gel dosimeters. Fricke gels rely on the radiation-induced oxidation of ferrous ions (Fe^2+^) to ferric ions (Fe^3+^), which also changes the gel’s magnetic properties. However, a major drawback of Fricke gels is the diffusion of these ions after irradiation, which blurs the initial dose distribution over time [20]. This makes them unsuitable for measuring complex, high-gradient dose distributions like those in MRT. Polymer gels, in contrast, form a solid polymer network at the site of irradiation, effectively “locking in” the dose distribution and minimizing signal diffusion, rendering them a more stable and accurate tool for high-resolution 3D dosimetry.

Gel dosimeters are produced from radiation-sensitive chemical constituents whose measurable properties vary in direct relation to the absorbed radiation dose [21]. The idea of using radiation-sensitive gels was introduced as early as the 1950s, when researchers demonstrated radiation-induced colour transformations in gels containing dyes such as methylene blue [22].

The concept of gel dosimetry includes several different types of well-described 3D dosimetry systems with a high spatial resolution [23]. All rely on a dose-dependent chemical substance change after irradiation, detectable by widely available readout systems such as magnetic resonance imaging (MRI) or optical-computerized tomography (optical-CT). A polymer gel is a hydrogel in which vinyl monomers are dispersed [24]. The polymer gel used in this study is a NIPAM gel, where the linear monomer, poly(N-isopropylacrylamide) is mixed with *N,N*′-methylene-bis-acrylamide as a monomer crosslinker. When exposed to ionizing radiation, the monomers undergo a radiation-induced polymerization reaction, which results in the formation of highly cross-linked, microscopical polymer clusters [23]. The degree of polymerization is related to the absorbed radiation dose. Within the gelatin matrix, the cross-linked microscopic polymer clusters are fixated spatially, and the absorbed dose is accumulated in a 3D pattern. During MRI read-out, the change in molecular mobility of the polymer has a significant effect on the transverse relaxation [24]. Thus, the acquired T2 value is significantly reduced by the created polymer. The calculated R2-values correspond to the accumulated relative absorbed dose in a 3D volume, with a spatial resolution in the order of micrometres.

Polymer gel dosimetry is a complete and independent detector system. The detector system’s response is unaffected by beam-angle incidence, field size, beam energy, and magnetic field strength [21]. The material is soft tissue-equivalent and can be moulded into various shapes [21]. Polymer gel detector systems have successfully been used in experimental radiotherapy previously, in the validation of novel radiotherapy techniques, to measure accumulated dose under conditions of anatomical deformation and as component of anthropomorphic dynamic phantoms [22,23,25].

The resolution limit of radiochromic films and gels is generally not the detector itself but rather the capability of the readout system. Our current work focuses on the still unmet need for 3D visualization and dosimetry in the development of MRT towards clinical application. We have explored NIPAM gels to analyse microbeam dose distributions and MRI-based readout for dose quantification. NIPAM gels have shown a good dose–response relationship [26]. The reported dose rate independence of NIPAM gels is advantageous for the varying, but typically high dose rates encountered in MRT [27], as it ensures accurate measurement across the entire dose rate spectrum. For irradiation doses higher than 2.8 Gy, a stabilization of the polymerization reaction can be achieved as early as 2 h after irradiation [28] which is especially attractive for clinical use where rapid results are often needed for an optimal workflow and quality assurance. While NIPAM gels are well established in broad beam dosimetry [29,30] and their MRI readout has been shown highly reproducible in different samples and batches [31], the capability of the gel to provide sufficient spatial resolution for microbeams has, to the best of our knowledge, not yet been explored.

We were able to show that the readout resolution of microbeams using NIPAM gel and MRI is comparable in quality to that of clinically established film dosimetry. This makes 3D rendering of dose distribution possible. The timely availability of these results, however, depends on access to high-field MR equipment and sufficient computational power for data processing. This work demonstrates that NIPAM gel dosimetry is a promising method for accurate high-resolution 3D dose verification in the development of MRT towards clinical applications.

## 2. Results and Discussion

### 2.1. Recording the Geometry of Microbeam Irradiation Patterns in NIPAM Gel

**Figure 1:**
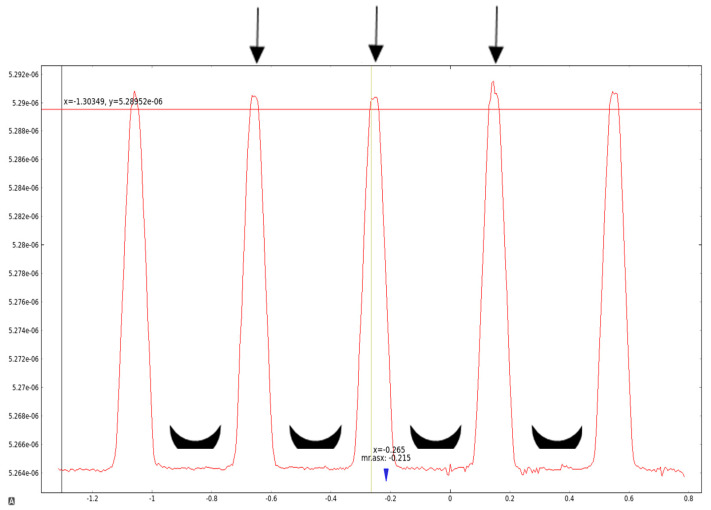
Microbeam profile at P61A. The arrows point to peak doses in the three central microbeams within the array of five, used to centre the collimator. The black crescents identify the valley dose zones created by the five microbeams. This plot shows real data collected using the X-Tream dosimeter (software version RADplot_20111111) at the P61A beamline using a 15 mm Au filter.

The irradiation experiment was carried out at the P61A beamline of the PETRA III/DESY synchrotron. Prior to sample irradiation, the multislit collimator (MSC) was aligned and the dose distribution profile within a single microbeam array was recorded. The incident beam at beamline P61A is relatively narrow (less than 2 mm), fitting 5 quasi-parallel, 50 µm wide microbeams spaced at a 400 µm centre-to-centre only (Figure 1).

**Figure 2:**
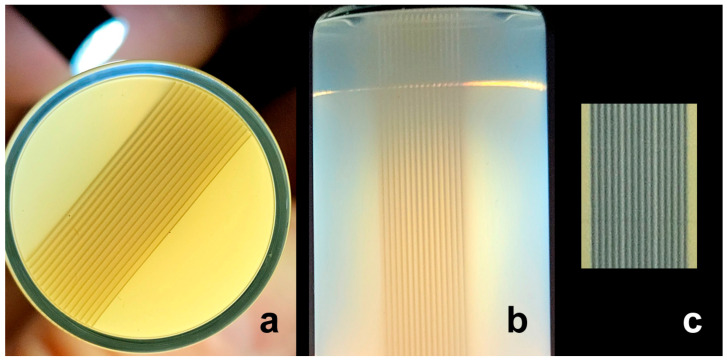
Unidirectional microbeam arrays visualized in the NIPAM gel, short axis (**a**) and long axis of the tube containing the gel (**b**) and recorded on EBT-3 Gafchromic™ film (**c**). In each case, the individual microbeams and the dose entry from scattered irradiation outside of the microbeam paths are clearly visible. The light source, shone against the glass vial in order to make the microbeams visible, can be seen in the left upper corner of (**a**). The inner diameter of the glass vial is 8 mm.

To increase the irradiation field laterally, we used three microbeam arrays, each containing five microbeams, which were laterally patched together. The resulting inhomogeneous dose distribution is recorded in NIPAM gel and on EBT-3 Gafchromic™ film (Ashland, Bridgewater, MA, USA). Each individual microbeam is clearly optically visible, both along the short and the long axes of the gel (Figure 2a,b) and on the radiochromic film (Figure 2c). A notable observation is the subtle darkening of the radiochromic material between the microbeams. This is not due to direct exposure to the microbeam, but rather by scattered irradiation that builds up the valley dose. The valley dose is a critical parameter, as it determines the potential for adverse effects on the surrounding normal, healthy tissue. The therapeutic advantage of MRT lies in the ability of normal tissue to repair the damage at peak doses as long as the valley dose is sufficiently low, and peaks are sufficiently narrow. Based on experience from clinical radiotherapy, we have determined a valley dose of approximately 10 Gy to be a suitable dose for microbeam studies. With a peak dose of 100 Gy and a valley dose of approx. 1.48 Gy in unidirectional MRT mode, the peak-to-valley dose ratio (PVDR) in our experiment was approximately 67.6. The PVDR, alongside the valley dose, is another important parameter determining the normal tissue tolerance. A higher PVDR indicates a steeper dose gradient between the high-dose peaks and the low-dose valleys. This translates into a lower risk of unwanted adverse effects in a clinical setting, which is a hallmark of improved quality of life for the patient.

In clinical radiotherapy, normofractionation is defined as 1.8–2.0 Gy per fraction. Hypofractionation, which involves the delivery of larger single fraction doses, offers the advantage of a higher biologically effective dose (BED) [32]. The extreme end of hypofractionated radiotherapy, known as radiosurgery (SRS), uses a single irradiation fraction. Such single fraction doses are typically used to treat smaller lesions in the brain, with target doses between 18.0 Gy and 24.0 Gy. The total target dose is split into smaller dose parcels entering the body from different angles called ports, and the radiation from each port converges at the intended irradiation target. This technique ensures that the normal tissue in the path of each beam is exposed to smaller doses, below its tolerance threshold, while the dose at the centre of the beam convergence is sufficiently high to destroy the tumour.

In our study, we have followed the concept of stereotactic coplanar irradiation. Since the position of the synchrotron beam is fixed, we rotated the irradiation target—the vials containing NIPAM gel—within the beam and irradiated from seven different angles (ports). Each port was created by laterally patching three microbeam arrays together, resulting in a total of 15 microbeams per port. In each of the 7 positions, the NIPAM gel was translated vertically through the horizontal synchrotron beam. The resulting valley dose was 10.7 Gy in the centre of the target (for peak doses of 100 Gy) with decreasing dose towards the periphery.

The diameter of the target can be enlarged by increasing the number of microbeam arrays to be patched laterally. However, this would also increase the overall irradiation time per sample. While this is not a significant issue for inanimate targets, longer irradiation times in living organisms might be problematic. From a radiobiological perspective, the outcome might depend not only on the administered dose but also on the overall irradiation time. Furthermore, in complex animals, physiological movements like breathing or heartbeats will cause tissue shifts which would lead to smearing of the microbeam edges and, consequently, a decrease in the PVDR. As the PVDR decreases, the risk of unwanted adverse effects might increase due to greater normal tissue damage. Therefore, to overcome this limitation, future in-vivo studies and clinical trials will require facilities that offer a wider treatment beam than the current PETRA III beamlines.

**Figure 3:**
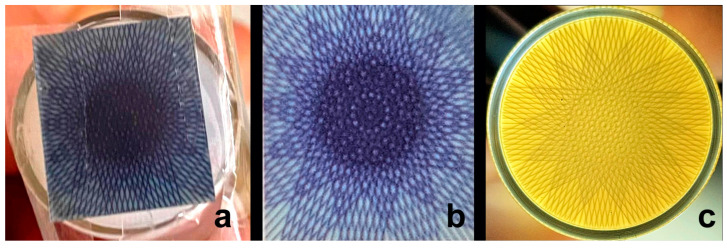
Multiport MRT, recorded on Gafchromic™ film taped to the bottom of the vial containing NIPAM gel (**a**), the film recovered from the vial (**b**) and in the NIPAM gel itself (**c**). The inner diameter of the glass vial is 15 mm.

The images in Figure 2a and Figure 3c were obtained by shining a light onto the wall of the glass vial containing the gel, perpendicularly to the direction of the microbeams. An optical CT system with a sufficiently high spatial resolution and penetration depth would be an optimal solution for 3D visualization of complex microbeam geometries. However, to the best of our knowledge, no such system is available that can meet the requirements for accurate microbeam dosimetry in larger volumes. In this study, the coplanar equiangular microbeam distribution creates a regular dose distribution pattern. In clinical practice, however, the beam geometry might look more irregular, due to the necessity of respecting dose constraints for organs at risk. For such cases, a true three-dimensional dose visualization would be even more important. Unlike a dedicated optical CT system, high-T MR scanners are much more readily available in research centres. We have, therefore, explored MRI as a tool for the analysis of three-dimensional dose distribution after MRT, as it is already validated for standard clinical dosimetry and provides a robust volumetric readout method that is not limited by the optical properties of the dosimeter.

### 2.2. MRI Analysis of NIPAM Gel Irradiated with Microbeams

The T2 relaxation time of the NIPAM gel is directly related to the absorbed radiation dose which causes chemical cross-linking of the polymers affecting T2-relaxation time. For a NIPAM gel dosimeter, the relevant physicochemical properties characterize the radiation-induced polymerization kinetics and the dosimetric response. This includes the polymer yield (G-value)**,** representing the number of monomer units polymerized per 100 eV of absorbed energy, and the dose sensitivity, typically quantified as the change in spin–spin relaxation rate (ΔR_2_) measured using MRI, which reflects the local polymerization induced by ionizing radiation. The G-value for NIPAM polymer gel dosimeters is typically in the range of 0.1–0.2 monomer units per 100 eV of absorbed energy, and ΔR_2_ is typically in the range of approx. 0.2–0.5 s^−1^ Gy^−1^.

**Figure 4:**
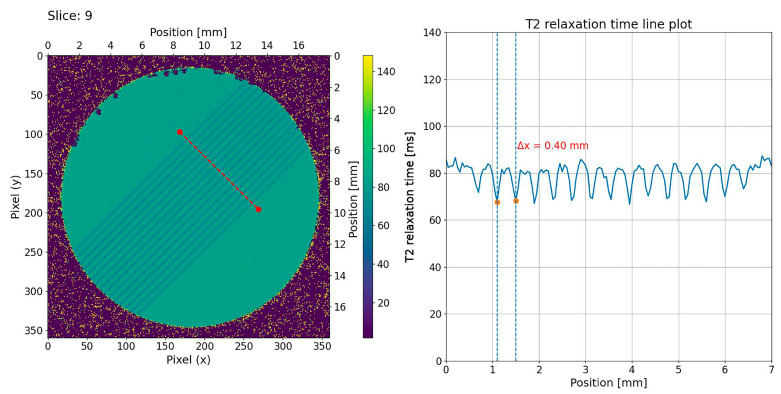
T2 relaxation map through a NIPAM gel exposed to unidirectional MRT. The centre-to-centre distance between the 50 µm wide microbeams is determined to be 400 µm using MRI images with an in-plane resolution of 50 × 50 µm.

**Figure 5:**
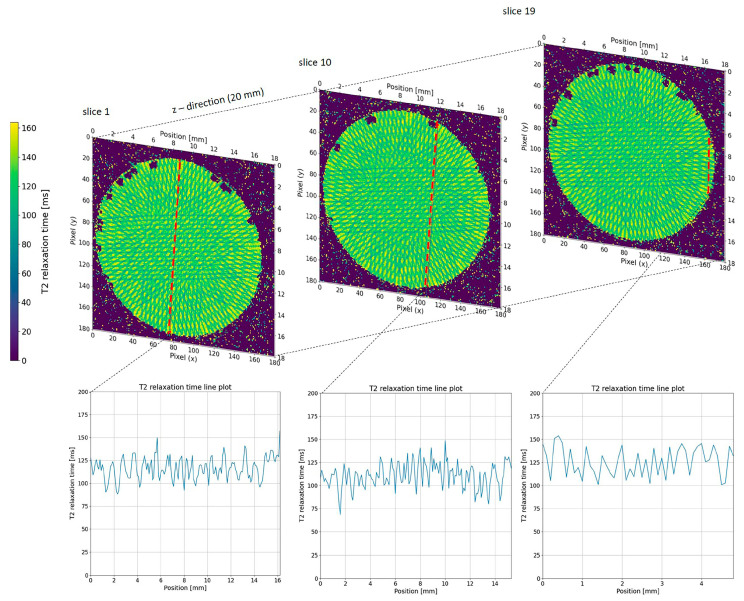
T_2_ relaxation time maps at three different z-positions of the NIPAM gel with three different line profiles along the indicated positions (red lines).

**Figure 6:**
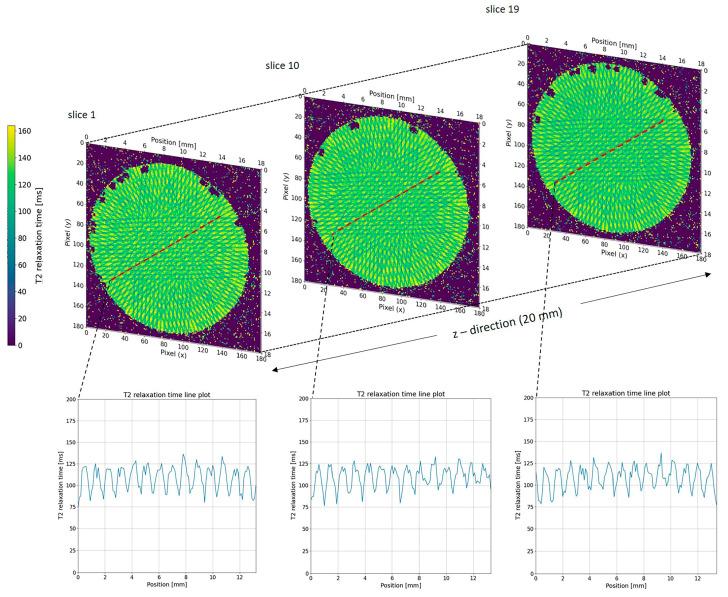
T_2_ relaxation time maps acquired at three different slice positions along the z-direction of the NIPAM gel. The red lines indicate the positions of the extracted line profile. The corresponding T_2_ relaxation time profile (right) shows the variation in T_2_ values along the selected line. The values are very similar for each xy position along the *z*-axis.

**Figure 7:**
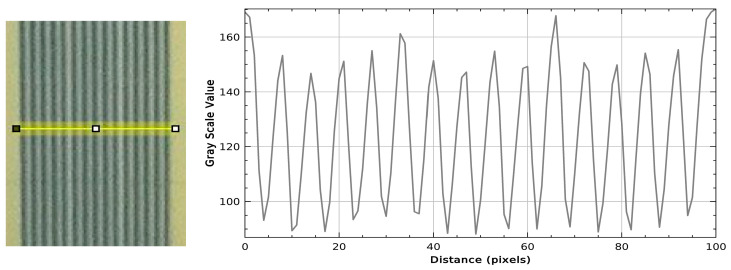
**G**rayscale values along the indicated line of the EBT-3 Gafchromic™ film.

We visualised calculated T2 relaxation maps using a custom Python script (version 3.11.13, Spyder IDE 6.0.8) to analyse the T2 relaxation time along a plotline for both single-port MRT (Figure 4) and multi-port MRT (Figure 5 and Figure 6). As a qualitative comparison we also analysed the grayscale values in the EBT-3 Gafchromic™ (Figure 7) using ImageJ software (ImageJ 1.53). With an MRI in-plane resolution of 100 × 100 µm, the microbeams can be visualized based on different T2 relaxation times across the microbeam array. By increasing the MRI in-plane resolution to 50 µm, we were able to achieve an acceptable signal-to-noise ratio (SNR) of 30 on average, though this raised the scanning time considerably. With this 50 µm resolution, we were able to accurately measure the centre-to-centre distance between the microbeams in both the line plot and the DICOM images (Figure 4), confirming the precise beam spacing. However, measuring the microbeam width itself in the MRI data is not possible as it would violate the sampling theorem, i.e., the MRI in-plane resolution of 50 µm is not smaller than the microbeam width of 50 µm. In order to achieve higher resolution T2 maps in a shorter time, an MRI system with higher field strength, steeper gradients, and a smaller coil diameter with higher SNR would be advantageous. However, even with these improvements it would be difficult to significantly overcome the spatial under sampling of the microbeam width, which remains a key limitation of this method.

While dose calculation in unidirectional MRT mode, as shown in Figure 4, is relatively straight forward, it becomes much more complicated in multiport MRT (Figure 6). However, despite the very complex beam geometry, the spatial resolution of the gel was sufficient to verify the resulting dose distribution pattern.

As an important aspect of data reproducibility (system reliability), we were able to show that equal positions within the geometric pattern created by the multiport irradiation returned similar relaxation values along the z-axis (Figure 7). This finding clearly demonstrates the reliability and consistency of both the NIPAM gel and MRI readout system.

The relatively low PVDR, seen in Figure 4, Figure 6 and Figure 7, is most likely due to non-linearity in the response of the NIPAM gel to higher radiation doses. A non-linear response has been noted in previous studies of NIPAM gel and is to be expected as the cross-linking reaction between the polymers in the gel will saturate at some dose. A similar saturation effect can be seen in the grayscale values of the EBT-3 Gafchromic™ film (Figure 7). It is possible that the dynamic range of the system can be increased by generating precise calibration curves or by using a higher field MR scanner and this will be the aim of future studies.

**Figure 8:**
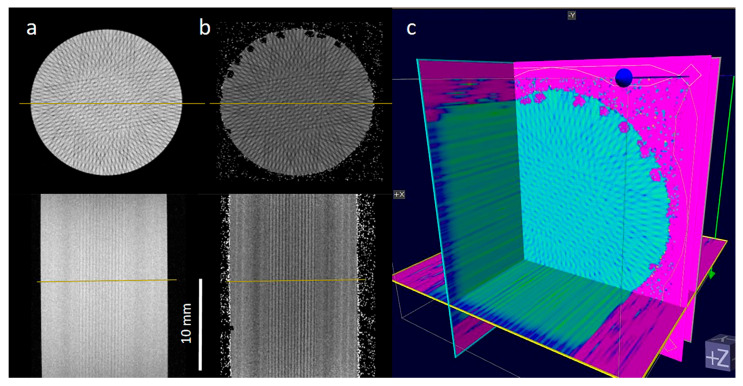
MRI images of NIPAM gel: Axial (**upper row**) and frontal (**lower row**) MRI images of NIPAM gel vials irradiated with a 7-port microbeam setup. (**a**) T_2_-weighted intensity images showing hypointense microbeam tracks; (**b**) corresponding calculated T_2_ relaxation time maps; (**c**) 3D rendering of the T_2_ relaxation time map.

From a clinical and radiobiological point of view, however, exact dosimetry of higher doses (above 20–40 Gy) is not a relevant parameter in determining the risk of normal tissue damage as this relies only on valley dose and preservation of field geometry, i.e., microbeam spacing. To this end, we were able to show that a three-dimensional rendering of the complex patterns created by multiport MRT, anticipated for future veterinary and human clinical trials, is possible (Figure 8).

## 3. Conclusions

Our results demonstrate that NIPAM gel holds strong potential as a three-dimensional dosimetry system for microbeam radiotherapy (MRT). By successfully capturing and visualising the geometric patterns of microbeam arrays with high-field MRI, we have established a clear proof of principle for this approach. The reliability of our system was confirmed through the reproducibility of measurements, and its validity was shown by the ability to accurately visualise complex dose distribution, a feature crucial for future clinical applications.

We acknowledge an important limitation in the system’s dynamic range, which is likely due to the non-linear response of the NIPAM gel at high doses above 20 Gy. This finding informs the direction of future work, which will focus on optimising the system through detailed calibration curves, readout optimization and variation in chemical composition of the NIPAM gel. By modifying the concentration of cross-linking compounds in the mixture, we would expect to increase the saturation threshold. Combining gel dosimeters with different dose sensitivity range could potentially yield a better estimation of PVDR. All data in this manuscript were obtained from a single batch of gel poured into multiple vials. As the aim of this study was to provide proof of principle, we did not carry out a detailed dose–response investigation. We would expect that each batch of gel would need to undergo an individual dose–response calibration to be reliable for quantitative dosimetry.

Our work shows that this method can produce an accurate three-dimensional rendering of the intricate dose patterns required for advanced MRT techniques, making it a promising tool for future veterinary and human trials.

## 4. Materials and Methods

### 4.1. NIPAM Gel Production

The NIPAM gel was prepared similar to the procedure described by Waldenberg and colleagues [33]. The gel was prepared under normal oxygen levels at ambient temperature under a fume hood. To avoid photopolymerization, the manufacturing process was performed in a relatively dark environment. Gelatin (Sigma Aldrich, Burlington, MA, USA, 5% *w*/*w*) was added to deionized water, which was heated to 45 °C to facilitate gelling. The suspension was stirred with a magnetic stirrer until the gelatin was completely dissolved. To the gelatin solution, first N-isopropyl acrylamide (NiPAM, 97% Sigma Aldrich, USA, 3% *w*/*w*), and second N,N’-methylene-bis-acrylamide (BIS, 98% Sigma Aldrich, USA, 3% *w*/*w*) were added. The solution was then cooled down to 38 °C and a solution of antioxidant tetrakis hydroxymethyl phosphonium chloride (THPC, 80% solution in water, Sigma Aldrich, USA) was added at 10 mM. The solution was continuously stirred until no solid components were seen anymore by visual inspection. The gel was then poured into glass vials with screw tops, left in a fridge at 8 °C overnight to solidify. The composition of the gel mixture is described in Table 1. The glass vials were 10 mL black cap Rotilabo (Roth, Karlsruhe, Germany) vials with an inner diameter of 15 mm, a total height of 60 mm and with an approximate fill height of 55 mm^2^). The data provided in this manuscript were obtained from the same batch of gel.

### 4.2. Microbeam Irradiation and Dosimetry

Microbeam irradiation was conducted at beamline P61A, the only white beam beamline of the PETRA III synchrotron on the DESY campus in Hamburg, Germany. The mobile insert module for biomedical research in the field of high-dose rate and spatially fractionated radiotherapy has been described previously [33]. The experiment was conducted at 40 bunch top-up mode, at a beam current of approx. 100 mA. All irradiation exposures were done in a window of 24–48 h after gel fabrication.

Prior to irradiation, the incident synchrotron beam was characterized using a flashDiamond detector Type 60025 (PTW, Freiburg, Germany), a synthetic diamond dosimeter specifically designed for high dose rate studies [34]. A multislit collimator (UNT, Morbier, France), described separately [35] was inserted in the incident beam to generate an array of five quasi-parallel microbeams. The individual microbeam width was 50 µm, spaced at a centre-to-centre distance of 400 µm.

Dose measurements and calibration were conducted using a silicon strip detector with the X-Tream dosimetry system (CMRP, Wollongong, Australia). The X-Tream system is a single channel electrometer with a high dynamic range, developed specifically for dosimetry in small volumes. It allows real-time read-out and can achieve a spatial dose resolution of approximately 6 µm [13]. It is, therefore, capable of resolving individual microbeams, determine peak-to-valley dose ratios and record dynamic MRT dose profiles [14]. For cross-calibration, PTW and X-Tream detectors were placed in a PMMA phantom at the same depth, using the same thickness of copper filters to modify the incident beam.

The measured suitable width of the incident synchrotron beam was 1.73 mm, just sufficient to accommodate five microbeams. In order to increase target width, a macro was developed, permitting the lateral patching of several microbeam arrays containing five microbeams each. Figure 2 shows an example of three laterally patched microbeam arrays, recorded on radiochromic film and in NIPAM gel.

The incident beam at the PETRA III synchrotron is typically only a few millimetres high. Furthermore, the position of the beam is fixed. To irradiate a sample higher than the beam height, the target needs to be moved vertically through the synchrotron beam. At any given dose rate, the speed of the vertical movement determines the dose deposited in the target. To laterally patch microbeam arrays, each vertical movement through the beam was followed by a lateral displacement of the sample. Three vertical movements and two lateral displacements of the sample were required to create a microbeam patch containing 15 microbeams. In our experiment, the incident beam was modified by a 13 mm Cu absorber, producing an average dose rate of approx. 601.87 Gy/s across all microbeams. The aperture-defining slits were set to produce a beam height of 1.38 mm at the sample position. The peak dose was 100 Gy and the peak-to-valley dose ratio (PVDR), one of the parameters defining normal tissue protection, was 67.49.

**Figure 9:**
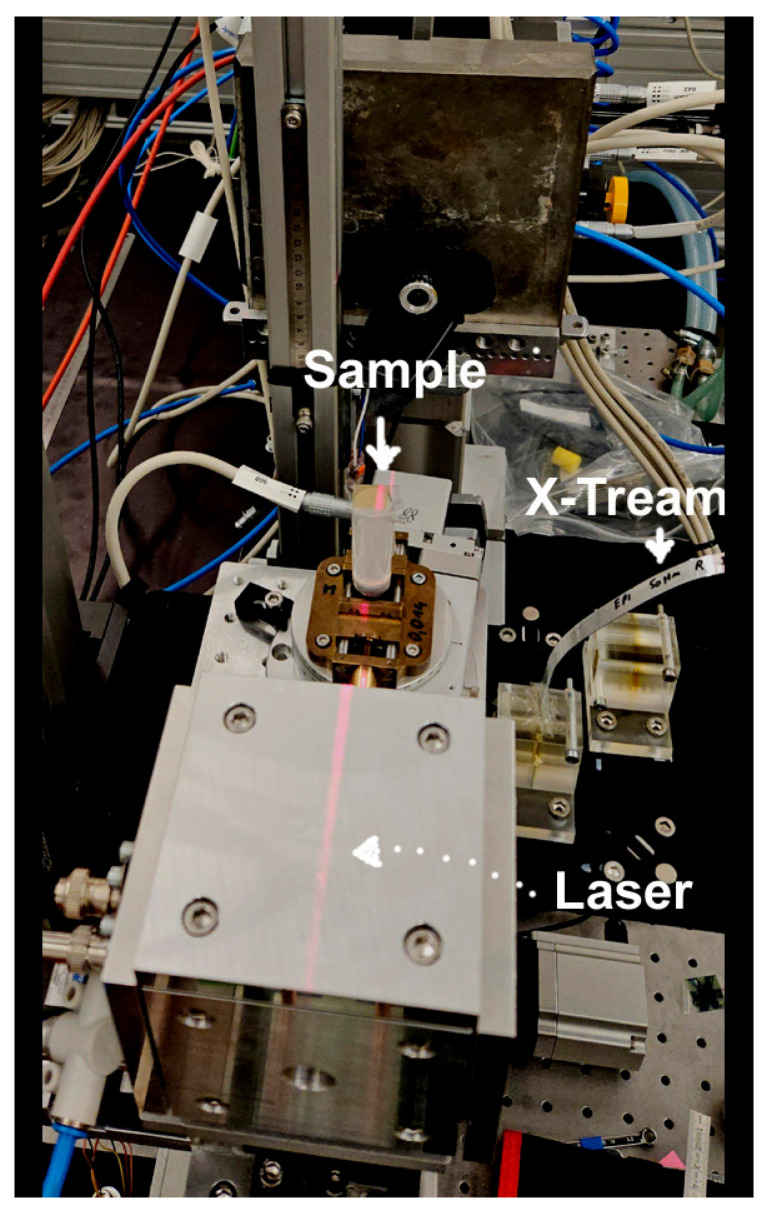
Experimental setup for microbeam irradiation of the NIPAM gels at the PETRA III beamline P61A. The red laser, marking the centre of the microbeam array, can be seen running atop of the MSC casing, hitting the glass vial containing the NIPAM gel approximately in mid-sample position.

To further improve dose coverage in the target, we aimed to irradiate in a coplanar stereotactic fashion, with dose entry from different angles (ports). We first determined the isocentre of the microbeam patch, located in the 8th of the 15 microbeams. For this proof-of-principle study, we have chosen an equiangular dose distribution with irradiation from 7 ports. Considering a PVDR of approx. 67.5, the maximum valley dose was approx. 10.37 Gy. The resulting geometric pattern was recorded on radiochromic film and in NIPAM gel, shown in Figure 3. The experimental setup is shown in Figure 9.

### 4.3. Magnetic Resonance Imaging of Gels After Irradiation

A preclinical 7 Tesla MRI scanner (BioSpec 70/30; BGA12S HP gradient system; gradient strength, 440 mT/m; Paravision software version 7.0, Bruker, Ettlingen, Germany) equipped with a 40 mm volume resonator (Model No. T13161V3, Bruker, Germany) was used to scan and read out the gel phantoms. T2w Multi-Slice Multi Echo (MSME) Sequences were used for T2-Mapping. The scan parameters are given in detail in Table 2. Following data acquisition, the T2-relaxation maps were fitted and calculated with the Bruker ISA-Tool in a pixel-by-pixel manner. A custom Python script was used for spatial and quantitative analysis of the calculated T2 relaxation time maps. All vials were scanned within a time window of 9 to 12 days post irradiation.

## Figures and Tables

**Table 1:** Composition of NIPAM-gel mixture.

**Sequence**	**Component**	**Amount**	**Temperature**
1	H_2_O, de-ionized	300 mL	ambient to
2	Gelatin	17 g	45 °C
3	NIPAM	10 g	45 °C
4	BIS	10 g	45 °C
5	THPC	1 mL	38 °C

**Table 2:** Technical parameters for scanning irradiated gels.

**Sequence Name**	**Repetition Time (TR)**	**Echo Time (TE)**	**Field of View (FoV)**	**Matrix**	**Resolution**	**Slice Number/Slice Thickness**	**Acq. Time**
T2-Map-MSME axial	2635 ms	12 Echos, spacing 7.5 ms (7.5–0 ms)	18 × 18 mm	180 × 180	100 × 100 µm	20/1 mm	31:37 min:s (4 Averages)
T2-Map-MSME axial (higher resolution)	2812 ms	12 Echos, spacing 10 ms (10–180 ms)	18 × 18 mm	360 × 360	50 × 50 µm	20/1 mm	8:26 hrs:s (30 Averages)
T2-Map-MSME frontal	3375.8 ms	12 Echos, spacing 15 ms (15–180 ms)	40 × 20 mm	180 × 180	100 × 100 µm	17/1 mm	31:37 min:s (4 Averages)

## Data Availability

All data are contained in the manuscript.

## Abbreviations

The following abbreviations are used in this manuscript:

MRI	Magnetic Resonance Imaging
MRT	Microbeam radiotherapy
NIPAM	N-isopropyl acrylamide
SFRT	Spatially fractionated radiotherapy
SNR	Signal-to-noise ratio
SRS	Stereotactic Radio Surgery
TE	Echo Time
TR	Repetition Time
